# Dietary phosphorus deficiency impaired growth, intestinal digestion and absorption function of meat ducks

**DOI:** 10.5713/ajas.18.0683

**Published:** 2019-04-15

**Authors:** Huimin Xu, Shujun Dai, Keying Zhang, Xuemei Ding, Shiping Bai, Jianping Wang, Huanwei Peng, Qiufeng Zeng

**Affiliations:** 1 Institute of Animal Nutrition, Key Laboratory for Animal Disease-Resistance Nutrition of China, Ministry of Education, Sichuan Agricultural University, Chengdu, Sichuan 611130, China

**Keywords:** Non-phytate Phosphorus, Growth Performance, Nutrient Utilization, Intestinal Morphology, Intestinal Enzyme Activity, Meat Duck

## Abstract

**Objective:**

An experiment was conducted to investigate the effects of dietary non-phytate phosphorus (nPP) deficiency on intestinal pH value, digestive enzyme activity, morphology, nutrient utilization, and gene expression of NaPi-IIb in meat ducks from 1 to 21 d of age.

**Methods:**

A total of 525 one-d-old Cherry Valley ducklings were fed diets (with 7 pens of 15 ducklings, or 105 total ducklings, on each diet) with five levels of nPP (0.22%, 0.34%, 0.40%, 0.46%, or 0.58%) for 21 d in a completely randomized design. Five experimental diets contained a constant calcium (Ca) content of approximately 0.9%. Body weight (BW), body weight gain (BWG), feed intake (FI), and feed to gain ratio (F:G) were measured at 14 and 21 d of age. Ducks were sampled for duodenum and jejunum digestion and absorption function on 14 and 21 d. Nutrient utilization was assessed using 25- to 27-d-old ducks.

**Results:**

The results showed ducks fed 0.22% nPP had lower (p<0.05) growth performance and nutrient utilization and higher (p<0.05) serum Ca content and alkaline phosphatase (ALP) activity. When dietary nPP levels were increased, BW (d 14 and 21), BWG and FI (all intervals), and the serum phosphorus (P) content linearly and quadratically increased (p<0.05); and the jejunal pH value (d 14), duodenal muscle layer thickness (d 14), excreta dry matter, crude protein, energy, Ca and total P utilization linearly increased (p<0.05); however, the serum ALP activity, jejunal Na^+^-K^+^-ATPase activity, and duodenal NaPi-IIb mRNA level (d 21) linearly decreased (p<0.05).

**Conclusion:**

The results indicated that ducks aged from 1 to 21 d fed diets with 0.22% nPP had poor growth performance related to poor intestinal digestion and absorption ability; but when fed diets with 0.40%, 0.46%, and 0.58% nPP, ducks presented a better growth performance, intestinal digestion and absorption function.

## INTRODUCTION

It is known that phosphorus (P) plays an important role in nucleic acid synthesis, energy metabolism, muscle function, enzyme activity, lipid metabolism and bone mineralization [1]. The effect of P on growth performance, bone development and bone quality in poultry has been well documented. Reduction of dietary non-phytate phosphorus (nPP) of broilers lead to poor bone mineralization and thus impaired animal welfare or increased processing losses [2]. Valable et al [3] also found that a reduction of 23% of dietary nPP content reduced bone mineral content and gain, breaking strength, tibia and toe ash weight in broilers from 1 to 21 d of age. Recently, some studies reported that P deficiency decreased the intestinal digestion and absorption ability in Jian carp, and impaired the intestinal immune barrier and physical barrier function in grass carp, resulting in a decrease in production performance [4,5]. Our previous study [6] also found that dietary nPP levels affected the diversity and structure of cecal microbiota in meat ducks from 1 to 21 d of age, more specifically finding that increasing the dietary nPP levels influenced the cecal microbiota and positively affected the growth of meat ducks. Ducks are more sensitive to diet P deficiency. Rodehutscord et al [7] found that Pekin ducks require less available P than the NRC [8] recommended based on the growth rate and P retention data. And the NRC [8] recommended that the nPP requirement of Pekin ducks for 1 to 14 d and 15 to 35 d was 0.40% and 0.30%, respectively. However, to our knowledge, there are no reports which evaluated the requirement of nPP based on the intestinal digestion and absorption ability in meat ducks.

A major challenge for poultry nutritionists in recent years [9] has been to decrease feed costs while maintaining bird performance at a high level with minimal environmental pollution. To overcome this problem, a comprehensive and better understanding of the intestinal digestion, absorption and utilization of P in poultry is needed. Digestion and absorption of nutrients in the intestine can be evaluated by many factors, such as pH value, digestive enzyme activity, and transporter carrying gene expression. For example, absorption processes of nutrients in the intestine are mainly driven by Na^+^-K^+^-ATPase that generates a Na^+^ and K^+^ concentration gradient and electric potential difference, allowing the absorption of various molecules [10]. The type IIb NaPi cotransporter (NaPi-IIb) is primarily expressed in the brush-border membranes of the duodenal epithelium, which is the major NaPi cotransporter and is responsible for active phosphate transport from the intestinal lumen into the blood [11]. It was reported that in the small intestine of chickens, the duodenum is the main site of absorption of active P, followed by jejunum and ileum, which suggests that differences in gene expression are associated with sites in the small intestine [12]. However, it remains unclear how dietary nPP levels regulate the pH value, digestive enzyme activity and gene expression of NaPi-IIb in the intestine of meat ducks. Therefore, the objective of this current study was to investigate the effect of dietary nPP levels on the intestinal digestion and absorption ability by determining the intestinal pH value, digestive enzyme activity, nutrient utilization, intestinal morphology and gene expression of NaPi-IIb in meat ducks from 1 to 21 d of age.

## MATERIALS AND METHODS

### Birds, diets and experimental design

The Institutional Animal Care and Use Committee of Sichuan Agricultural University approved all procedures used in this study. A total of 525 1-d-old male Cherry Valley ducks (Anas platyrhynchos) were obtained from a commercial hatchery (Mianying duck breeding farm, Sichuan Province, P. R. China), and randomly divided into one of five dietary treatments, each with seven replicate pens of 15 ducks, in a completely randomized design for 21d. Five experimental diets were formulated to contain 0.22%, 0.34%, 0.40%, 0.46%, and 0.58% nPP, supplemented with 0.09%, 0.21%, 0.27%, 0.33%, and 0.45% of inorganic P in the form of monocalcium phosphate (CaH_2_PO_4_·2H_2_O), respectively. The analyzed total P (TP) content in the experimental diets was 0.54%, 0.72%, 0.79%, 0.84%, or 0.92%, respectively. Each diet contained a constant calcium content of approximately 0.90%. Therefore, the Ca:nPP in the five experimental diets was 4.09:1, 2.65:1, 2.25:1, 1.96:1, and 1.55:1, respectively.

The basal diet (1 to 21 d) (Table 1, 2-mm-diameter-pellet) was formulated under a digestible amino acid basis to meet the nutrient requirements of Pekin ducks suggested in the NRC [8] and Han et al [13] except for P. Ducks were reared in pens (2.2 m×1.2 m×0.9 m) in a temperature- and humidity-controlled room with a 24-h constant light schedule and free access to water and feed.

### Data collection and sampling

At the end of the experiment, and after 12 h of feed withdrawal, ducks were weighed, and feed consumption was obtained for each pen. Body weight (BW) and body weight gain (BWG), feed intake (FI), and feed to gain ratio (F:G) were calculated accordingly at the following intervals: 1 to 14 d, 15 to 21 d, and 1 to 21 d. Feed waste was recorded daily, and the data were used in the calculations of feed consumption. Birds that died during the experiment were weighed, and the data were used in the calculations of F:G.

Then, on d 14 and 21, one duck per pen with a weight clos est to the pen average was selected and bled from the jugular vein, respectively. The blood samples (n = 7) were centrifuged at 3,000 g/15 min at 4°C, and serum was collected and stored at −20°C for Ca, P content and alkaline phosphatase (ALP) activity determination. After that, all ducks were euthanized by cervical dislocation. The duodenal and jejunal segments were quickly isolated and flushed with ice cold saline solution. Then, the duodena land jejuna mucosa (n = 7) were scraped off with an ice-cold microscope slide, immediately frozen and stored in liquid nitrogen for assay of NaPi-IIb gene expression. Then, the digestive contents of the jejunum (n = 7) were collected by gently squeezing and stored at −20°C for digestible enzyme activities. The acidity of the digesta in gizzard, duodenum, jejunum, ileum and cecum was immediately measured by direct insertion of a pH electrodes indicated by Liao et al [14]. After collecting digesta, 1.5-cm sections of mid duodenum and jejunum were separated for morphological assessment. The procedures and equipment used were the same as described by Han et al [13].

### Serum Ca, P concentration and alkaline phosphatase activity

Serum Ca and P concentrations were analyzed using a Bio-chemistry Analyzer (Yellow Springs Instrument Co., Inc., Yellow Springs, OH, USA). The serum’s ALP activity was determined by conversion of p-nitrophenyl phosphate to p-nitrophenol and monitored at 405 nm using a Gemini XPS Microplate Reader (Molecular Devices, LLC., Sunnyvale, CA, USA) as indicated by Zhang et al [15]. The kits in this trial were obtained from Nanjing Jiancheng Bioengineering Institute (Nanjing, Jiangsu, China).

### Jejunal enzyme activity

Enzyme activities of ALP and Na^+^-K^+^-ATPase in digesta from the jejunum were determined according to the analytical kits (Nanjing Jiancheng Bioengineering Institute, China) after digesta was homogenized with a cold medium (weight:volume = 1:9), provided by the kit, in an ice-water bath according to Zhu et al [16] and Liu et al [17].

### RNA isolations and real-time polymerase chain reaction

The RNA isolation and real-time polymerase chain reaction (PCR) were performed as previously described in our laboratory [18]. Briefly, total RNA was isolated from the duodenal and jejunal mucosa by using a TRIZOL reagent (Invitrogen, Carlsbad, CA, USA) according to the manufacturer’s instructions. The RNA concentration was measured by using the Nano Drop ND-1000 spectrofluorometer (Nano-Drop Technologies, Wilmington, DE, USA), and the quality of total RNA was determined in agarose gels stained with ethidium bromide. One microgram of total RNA was subjected to reverse transcription with the Super Script First-Strand Synthesis System (Invitrogen, USA). Real-time PCR reactions were performed on an ABI 7500 real-time PCR system using SYBR Green PCR Master Mix (Applied Biosystems, Foster City, CA, USA). The primer sequences for NaPi-IIb mRNA were GG TAAAGCAGCAGGGGACAT (Forward) and CTGAGGT GCCAATGTTTGCC (Reverse). The primer sequences for β-actin (housekeeping gene) mRNA were AAGTACCCCATT GAACACGGT (Forward) and TCTGTTGGCTTTGGGG TTCA (Reverse). Gene-specific amplification was determined by melting curve analysis and agarose gel electrophoresis. Relative quantities of mRNA were calculated using the 2^−ΔΔCt^ method [19], and values were normalized by β-actin as an internal control.

### Metabolic study

On d 22, two birds per pen were randomly selected (14 ducks per treatment, 70 ducks in total) and transferred to metabolic cages (two ducks per cage) and fed with the original starter diets mixed with chromic oxide (0.3%). After a three-day adaptive period (d 22, 23, and 24), the total excreta samples from each cage were collected for 72 hours (d 25, 26, and 27). Excreta were weighed and then stored at −20°C immediately. These excreta were dried at 65°C±5°C for 24 h, weighed and crushed to pass through a 40-mesh sieve for dry matter (DM), Cr, N, ether extract and energy availability according to Adeola [20] and Zeng et al [21].

### Statistical analyses

All data were analyzed using SAS statistical software (version 9.2, SAS Institute Inc., Cary, NC, USA), and data were analyzed based on methods described by Seo et al [22]. The experimental unit was the replicate pen (n = 7) for growth performance, and an individual duck (n = 7) for gizzard and intestinal characteristics measurements at 14 and 21 d of age, respectively. The effect of dietary nPP levels was determined by performing a one-way analysis of variance using the general linear model procedure in SAS software (SAS Institute Inc., USA). When the dietary effect was significant (p<0.05), polynomial contrasts and the linearity of the response to analyzed dietary nPP levels were examined using linear and quadratic regression. The R^2^ value was provided to compare these regressions when the linear or quadratic effect was significant (p<0.05) [23]. Probability values ≤0.05 were considered significant, and those <0.1 but >0.05 were considered to show a tendency towards approaching significance.

## RESULTS

The chemical values of Ca and TP in diets were close to expected values, confirming that the ingredients were mixed correctly.

### Growth performance

Dietary nPP levels linearly and quadratically increased (p< 0.05, Table 2) BW (d 14 and 21), as well as BWG and FI (all intervals), but they had no effect on F:G. Ducks fed the diet with 0.22% nPP had the lowest (p<0.05) BW, BWG, and FI in all intervals compared to ducks fed the other four diets. In addition, when compared to the 0.46% nPP group, birds fed the diet containing 0.34% nPP had lower (p<0.05) BW at 14 d of age and BWG during 1 to 14 d of age. From 1 to 21 d of age, F:G showed a linear (p = 0.079) and quadratic (p = 0.061) decrease as dietary nPP increased.

### Serum parameters

As shown Table 3, dietary nPP levels linearly and quadratically decreased (p<0.05) the serum Ca content (d 14 and 21), and linearly and quadratically increased (p<0.05) the serum P content (d 14). With the increase of dietary nPP levels, serum ALP activity at 14 d of age presented a linear and quadratic decrease (p<0.05), but at 21 d of age, it showed a linear decrease (p<0.05). Ducks fed the diet containing 0.22% nPP had a higher (p<0.05) serum Ca content and ALP activity and lower (p<0.05) serum P content.

### Intestinal pH value, digestive enzyme activity and morphology

Data for the gizzard and intestinal pH value are presented in Table 4. In general, dietary nPP levels had no effect on the pH value in the gizzard, duodenum (d 21), jejunum (d 21), ileum (d 14) and cecum. With the increase in dietary nPP levels, the pH value linearly increased (p<0.05) in the jejunum (d 14), showed a tendency towards increasing (p = 0.071) in the duodenum (d 14), and showed a tendency towards quadratically increasing (p = 0.072) in the ileum (d 21).

With an increase in dietary nPP levels, jejunal Na ^+^-K^+^-ATPase activity linearly decreased (p<0.05, Table 5) at 14 d of age and showed a tendency towards decreasing (p = 0.058) at 21 d of age. However, dietary nPP levels had no effect on jejunal ALP activity.

Dietary nPP levels presented a linear effect (p <0.05, Table 6) on the duodenal muscle layer thickness (d 14) and showed a tendency towards increasing the duodenal villus height (p = 0.077). Dietary nPP levels had no effect on the other morphologic duodenal parameters and jejunal morphology.

### Excreta nutrient utilization

The effects of dietary nPP levels on excreta nutrient utilization are shown in Table 7. The increase of dietary nPP levels linearly increased (p<0.05) the excreta DM, crude protein, energy, Ca and TP utilization. In general, the diet containing 0.58% nPP had better excreta nutrient utilization and higher (p<0.05) DM, energy, Ca, and TP availability compared with the diets containing 0.22%, 0.34%, and 0.40% nPP.

### Intestinal NaPi-IIb gene expression

**Figure 1:**
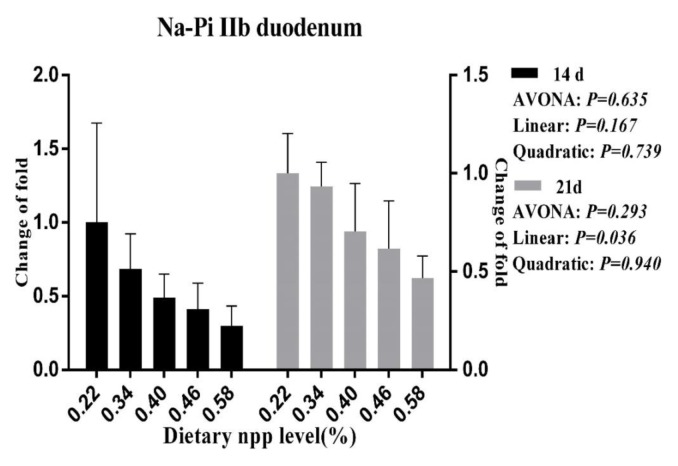
Effect of dietary non-phytate phosphorous levels on NaPi-IIb gene expression in duodenum of ducks at 14 d and 21 d of age. NaPi-IIb, type IIb sodium-phosphate cotransporter; npp, non-phytate phosphorous.

**Figure 2:**
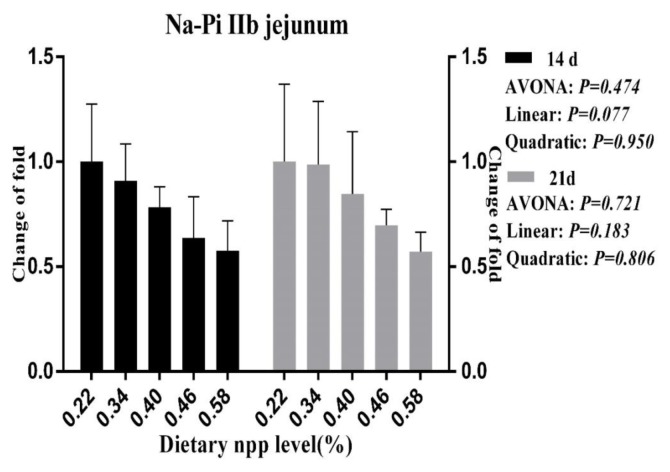
Effect of dietary non-phytate phosphorous levels on NaPi-IIb gene expression in jejunum of ducks at 14 d and 21 d of age. NaPi-IIb, type IIb sodium-phosphate cotransporter; npp, non-phytate phosphorous.

As the dietary nPP levels increased, a linear (p<0.05, Figure 1) decrease was observed for the NaPi-IIb mRNA level in the duodenum at 21 d of age, and a tendency towards decreasing (p = 0.077, Figure 2) was observed for the NaPi-IIb mRNA level in the jejunum at 14 d of age.

## DISCUSSION

In the current study, a 0.34% level of nPP in the diet reduced BW (14 d) and BWG (d 1 to 14), and a lower dietary nPP level (0.22%) further decreased the growth performance in ducks aged 1 to 21 d. These results suggest that the nPP requirement of ducks decreases as the ducks’ age increases. Ducks fed diet with 0.40% nPP presented a normal performance. The dietary 0.40% nPP level is in line with the NRC [8] recommended, and which agreed with Xie et al [24] who studied the interaction between dietary Ca and nPP on growth performance and bone ash in Pekin ducklings, and they used a quadratic regression to predict that the requirements of Ca and nPP for maximum weight gain and minimum feed/gain for ducks aged from 1 to 14 d were 0.806% and 0.403%, 0.796% and 0.379%, respectively.

One reason for the decrease of FI and growth performance that occurs when ducks are fed a diet with a deficiency in P, and in the present experiment those that were fed the diets with 0.22% and 0.34% nPP was that these two diets had a higher Ca:nPP (4.09:1 and 2.65:1). Hulan et al [25] found that the dietary Ca:nPP above 2.81:1 could depress the growth performance and FI in broilers. And Rama Rao et al [26] also observed that broilers fed diets with 2.3–3:1 Ca:nPP had poor performance. Because the two minerals tend to form calcium phosphate, an insoluble complex in the chicken gut resulting in reduced absorption, which may be the reason for growth retardation at higher Ca:nPP [27]. Another reason might be due to differences in dietary Ca and metabolizable energy levels, as well as the types of basal diet. Zeng et al [21] reported that dietary metabolizable energy concentration could affect the dietary nPP level by regulating the FI of ducks. A further reason for the decrease of FI and growth performance that occurs when ducks are fed a diet with a deficiency in P, is that their gut microbial communities need to obtain P from the degradation of phytate [6], requiring that more energy is spent on the maintenance of the basic metabolism, and less energy is available to spend on nutrient utilization and growth.

The important finding of the present study was that as the dietary nPP level increased, the serum ALP activity, jejunal Na^+^-K^+^-ATPase activity, and NaPi-IIb mRNA expression linearly decreased, while the intestinal morphology and nutrient utilization linearly increased, especially at 14 d of age, indicating that dietary nPP deficiency impaired the intestinal digestion and absorption ability of ducks. Because Manzanilla et al [28] and Hou et al [29] reported that the intestinal villus height and the crypt depth were closely related to intestinal absorption ability, the shallower crypt depth and the higher villus height indicated that intestinal tract had a better absorption ability. In this study, the villus height of duodenum and jejunum in 0.46% nPP group increased by 14.19% and 5.52%, and 9.50% and 1.02% compared with 0.22% nPP group at 14 and 21 d of age, respectively. Emami et al [30] also stated that birds fed a P deficient diet had a shorter villus height, a higher crypt depth, and a lower villus height/crypt depth ratio than birds fed the adequate amount of available phosphorus diet in broiler. In the current study, we found ducks fed diet with 0.46% and 0.58% nPP had the best nutrient utilization when compared to the other 3 diets, which suggested that duck need more nPP to maintain a better intestinal digestion and absorption function. This result agreed with Dai et al [6], who found ducks fed diets with 0.46% and 0.58% nPP had a better cecal microbiota diversity and composition.

Rhoads et al [31] reported that the activity of Na ^+^-K^+^-ATPase indirectly reflected the absorption function of intestinal mucosa, and ATPase was required to provide energy in active absorption. It is reported that the activity of Na^+^-K^+^-ATPase in the lower intestine was significantly reduced in Wistar rats fed diets with added phytic acid compared with controls [32]. The decrease in activity of Na^+^-K^+^-ATPase may be related with the increase of pH value in the intestine. In this current study, we observed that when dietary inorganic P supplementation increased, the pH values in the jejunum, duodenum and ileum tended to increase. Intestinal pH values may be considered when using inorganic P sources to increase dietary nPP levels because gastrointestinal tract acidity is considered an important factor that affects the digestive environment [33]. Capuano et al [34] reported that the manipulation of dietary pH to produce a lower gastric and intestinal pH may be beneficial for digestion and for the maintenance of more desirable microflora.

Moreover, some researchers have observed that NaPi-IIb expression was up-regulated under conditions of dietary P deprivation [35]. When dietary nPP was increased, the NaPi-IIb expression levels were reduced in our study and in studies with broilers [10,11], laying hens [36] and mice [32]; this outcome indicates that the duodenal P absorption capacity could be decreased with increasing serum P levels. In the present study, we also observed consistently that serum P levels increased as dietary nPP levels increased. Therefore, both the present experiment and the previous researches indicate that ducks might regulate to increase P absorption in the small intestine when dietary nPP is lower than the nPP requirement. Increasing active absorption of P under a lower level of dietary nPP, means that more energy was consumed, which leads to the decrease of nutrient utilization and growth performance.

In summary, a 0.22% level of nPP in the diet of ducks aged from 1 to 21 d reduced growth performance and nutrient utilization, damaged the intestinal morphology, and increased intestinal Na^+^-K^+^-ATPase activity and NaPi-IIb mRNA levels, which indicated a poor intestinal digestion and absorption function. Increasing dietary nPP levels could increase the growth performance of meat ducks via improving the intestinal digestion and absorption ability, and ducks aged from 1 to 21 d fed diets with 0.40%, 0.46%, and 0.58% nPP presented a better growth performance, and a better intestinal digestion and absorption ability.

## Figures and Tables

**Table 1:** Ingredients and compositions of the experimental diets (%, dry matter basis)

Ingredients	Diet 1	Diet 2	Diet 3	Diet 4	Diet 5
Corn	59.50	59.50	59.50	59.50	59.50
Soybean oil	1.87	1.87	1.87	1.87	1.87
Soybean meal	33.85	33.85	33.85	33.85	33.85
L-lysine-HCL	0.024	0.024	0.024	0.024	0.024
DL-methionine	0.197	0.197	0.197	0.197	0.197
Limestone	1.905	1.710	1.613	1.516	1.320
Monocalcium phosphate	0.395	0.928	1.196	1.465	2.001
Tryptophan	0.061	0.061	0.061	0.061	0.061
Bentonite	1.169	0.831	0.660	0.488	0.150
Sodium chloride	0.35	0.35	0.35	0.35	0.35
Choline chloride	0.15	0.15	0.15	0.15	0.15
Vitamin premix1)	0.03	0.03	0.03	0.03	0.03
Mineral premix2)	0.50	0.50	0.50	0.50	0.50
Total	100.00	100.00	100.00	100.00	100.00
Calculated nutrients levels (%)					
Metabolizable energy (MJ/kg)	12.12	12.12	12.12	12.12	12.12
Crude protein	19.5	19.5	19.5	19.5	19.5
Calcium	0.90	0.90	0.90	0.90	0.90
Non-phytate phosphorus	0.22	0.34	0.40	0.46	0.58
Digestible lysine	0.96	0.96	0.96	0.96	0.96
Digestible methionine	0.46	0.46	0.46	0.46	0.46
Digestible threonine	0.66	0.66	0.66	0.66	0.66
Digestible tryptophan	0.26	0.26	0.26	0.26	0.26
Analyzed nutrients concentration					
Total phosphorus (%)	0.54	0.72	0.79	0.84	0.92
Calcium (%)	0.93	0.94	0.92	0.94	0.95

1) Provided per kilogram of diet vitamin A, 8,000 IU; cholecalciferol, 2,000 IU; vitamin E, 5 IU; vitamin K, 1 mg; thiamine, 0.4 mg; riboflavin, 3.2 mg; pyridoxine, 1.2 mg; vitamin B_12_, 6 μg; folic acid, 100 μg; niacin, 7 mg; calcium pantothenate, 5 mg.

2) Provided per kilogram of diet: Fe (FeSO_4_·H_2_O) 80 mg, Cu (CuSO_4_·5H_2_O) 8 mg, Mn (MnSO_4_·H_2_O) 70 mg, Zn (ZnSO_4_·H_2_O) 90 mg, I (KI) 0.4 mg, Se (Na_2_SeO_3_) 0.3 mg.

**Table 2:** Effect of dietary non-phytate phosphorus levels on growth performance of ducks

Index	Dietary non-phytate phosphorus level (%)					SEM	p-value		
	
	0.22	0.34	0.40	0.46	0.58		ANOVA	Linear	Quadratic
Body weight (g)									
1 d1)	57.4	57.4	57.4	57.4	57.3	0.05	0.949	0.562	0.716
14 d1)	745^c^	852^b^	875^ab^	887^a^	865^ab^	11.94	<0.01	<0.01	<0.01
21 d2)	1,310^b^	1,498^a^	1,505^a^	1,540^a^	1,505^a^	17.90	<0.01	<0.01	<0.01
Body weight gain (g)									
1–14 d	687^c^	795^b^	818^ab^	830^a^	808^ab^	11.95	<0.01	<0.01	<0.01
15–21 d	566^b^	646^a^	630^a^	653^a^	640^a^	12.73	0.00	0.00	0.002
1–21 d	1,253^b^	1,441^a^	1,448^a^	1,483^a^	1,448^a^	17.90	<0.01	<0.01	<0.01
Feed intake (g)									
1–14 d	849^b^	965^a^	1,002^a^	996^a^	988^a^	21.69	<0.01	<0.01	0.001
15–21 d	999^b^	1,108^a^	1,093^a^	1,117^a^	1,106^a^	19.94	0.001	0.001	0.008
1–21 d	2,158^b^	2,405^a^	2,448^a^	2,454^a^	2,435^a^	27.59	<0.01	<0.01	<0.01
Feed to gain ratio									
1–14 d	1.23	1.21	1.23	1.20	1.22	0.02	0.803	0.637	0.447
15–21 d	1.77	1.72	1.74	1.71	1.73	0.04	0.865	0.499	0.502
1–21 d	1.72	1.67	1.69	1.65	1.68	0.02	0.066	0.079	0.061

SEM, standard error of the mean; ANOVA, analysis of variance.

1) Means represent 7 pens of 15 ducks per pen.

2) Means represent 7 pens of 14 ducks per pen.

a–c Means in the same row with no common superscript are significantly different (p<0.05).

**Table 3:** Effect of dietary non-phytate phosphorus levels on the serum parameters of ducks

Index	Dietary non-phytate phosphorus level (%)					SEM	p-value		
	
	0.22	0.34	0.40	0.46	0.58		ANOVA	Linear	Quadratic
Serum calcium (mmol/L)									
14 d1)	2.68^a^	1.86^b^	1.94^b^	1.94^b^	1.98^b^	0.06	<0.01	<0.01	<0.01
21 d	2.92^a^	2.30^b^	2.28^b^	2.28^b^	2.20^b^	0.13	0.002	0.001	0.025
Serum phosphorus (mmol/L)									
14 d	1.26^b^	1.98^a^	1.87^a^	1.87^a^	1.84^a^	0.08	<0.01	0.000	<0.01
21 d	1.56	2.13	2.10	2.00	1.98	0.19	0.240	0.193	0.086
Alkaline phosphatase (K/100 mL)									
14 d	1,326^a^	792^bc^	898^b^	707^c^	734^c^	42.7	<0.01	<0.01	<0.01
21 d	1,211^a^	872^b^	770^b^	774^b^	752^b^	105	0.021	0.004	0.071

SEM, standard error of the mean; ANOVA, analysis of variance.

1) Means represent 7 pens of 1 duck per pen.

a–c Means in the same row with no common superscript are significantly different (p<0.05).

**Table 4:** Effect of dietary non-phytate phosphorus levels on gastrointestinal pH of ducks

Index	Dietary non-phytate phosphorus level (%)					SEM	p-value		
	
	0.22	0.34	0.40	0.46	0.58		ANOVA	Linear	Quadratic
Gizzard									
14 d1)	3.74	3.96	4.17	3.27	3.53	0.29	0.222	0.312	0.441
21 d	3.25	2.95	3.05	3.38	3.31	0.26	0.737	0.605	0.495
Duodenum									
14 d	6.20	6.50	6.42	6.41	6.47	0.09	0.145	0.071	0.199
21 d	6.51	6.19	6.41	6.30	6.33	0.10	0.280	0.357	0.244
Jejunum									
14 d	6.36	6.43	6.48	6.45	6.60	0.07	0.238	0.029	0.760
21 d	6.37	6.20	6.35	6.30	6.16	0.10	0.478	0.238	0.765
Ileum									
14 d	7.08	6.94	6.96	7.03	7.05	0.14	0.943	0.971	0.482
21 d	6.73	7.04	7.24	6.76	6.73	0.18	0.192	0.721	0.072
Cecum									
14 d	6.38	6.55	6.82	6.51	6.53	0.22	0.711	0.675	0.353
21d	5.78	5.58	5.79	5.92	5.81	0.18	0.763	0.596	0.878

SEM, standard error of the mean; ANOVA, analysis of variance.

1) Means represent 7 pens of 1 duck per pen.

**Table 5:** Effect of dietary non-phytate phosphorus levels on jejunal enzyme activity of ducks

Index	Dietary non-phytate phosphorus level (%)					SEM	p-value		
	
	0.22	0.34	0.40	0.46	0.58		ANOVA	Linear	Quadratic
Alkaline phosphatase (U/mgprot)									
14 d1)	930	931	899	901	894	18.57	0.464	0.113	0.887
21 d	933	936	921	923	921	14.68	0.921	0.467	0.928
Na^+^-K^+^-ATPase (U/mgprot)									
14 d	42	42	41	40	39	0.86	0.265	0.035	0.750
21 d	43	42	40	39	40	1.53	0.300	0.058	0.486

SEM, standard error of the mean; ANOVA, analysis of variance.

1) Means represent 7 pens of 1 duck per pen.

**Table 6:** Effect of dietary non-phytate phosphorus levels onintestinal morphology of ducks

Index	Dietary non-phytate phosphorus level (%)					SEM	p-value		
	
	0.22	0.34	0.40	0.46	0.58		ANOVA	Linear	Quadratic
Duodenum									
Villus height (μm)									
14 d1)	628	635	682	717	732	48.93	0.459	0.077	0.973
21 d	594	599	602	627	617	51.19	0.990	0.671	0.953
Crypt depth (μm)									
14 d	136	149	154	149	153	9.42	0.670	0.229	0.444
21 d	148	135	156	150	142	8.61	0.504	0.991	0.745
Villus height/crypt depth									
14 d	4.72	4.48	4.65	4.85	4.87	0.37	0.948	0.632	0.696
21 d	4.16	4.48	3.98	4.48	4.57	0.44	0.856	0.527	0.840
Muscle layer thickness (μm)									
14 d	261	290	302	356	335	25.72	0.091	0.015	0.484
21 d	357	320	313	325	333	16.28	0.374	0.346	0.086
Jejunum									
Villus height (μm)									
14 d	516	587	604	565	552	37.67	0.537	0.607	0.133
21 d	530	578	511	535	554	29.71	0.569	0.834	0.904
Crypt depth (μm)									
14 d	138	142	125	133	133	7.87	0.645	0.519	0.656
21 d	127	128	136	136	126	9.11	0.886	0.928	0.411
Villus height/crypt depth									
14 d	3.83	4.26	4.97	4.38	4.43	0.39	0.353	0.266	0.208
21 d	4.56	4.75	3.84	4.19	4.78	0.45	0.540	0.968	0.284
Muscle layer thickness (μm)									
14 d	271	284	272	298	280	18.17	0.825	0.600	0.652
21 d	265	287	291	308	292	15.49	0.420	0.148	0.258

SEM, standard error of the mean; ANOVA, analysis of variance.

1) Means represent 7 pens of 1 duck per pen.

**Table 7:** Effect of dietary non-phytate phosphorous levels on excreta nutrient utilization of ducks

Index	Dietary non-phytate phosphorus level (%)					SEM	p-value		
	
	0.22	0.34	0.40	0.46	0.58		ANOVA	Linear	Quadratic
DM (%)	67^c^	68^c^	72^b^	73^ab^	74^a^	0.72	<0.01	<0.01	0.516
CP (%)	65^b^	56^c^	66^ab^	64^b^	68^a^	1.02	<0.01	0.001	0.000
Energy (%)	73^c^	73^c^	76^b^	77^ab^	78^a^	0.61	<0.01	<0.01	0.935
Ca (%)	51^c^	49^c^	55^b^	66^a^	65^a^	1.48	<0.01	<0.01	0.410
TP (%)	35^d^	46^bc^	51^b^	45^c^	59^a^	2.39	<0.01	<0.01	0.775

SEM, standard error of the mean; ANOVA, analysis of variance; DM, dry matter; CP, crude protein; Ca, calcium; TP, totalphosphorus.

a–d Means in the same row with no common superscript are significantly different (p<0.05).
